# A five-pseudouridylation-associated-LncRNA classifier for primary prostate cancer prognosis prediction

**DOI:** 10.3389/fgene.2022.1110799

**Published:** 2023-01-10

**Authors:** Pengxiang Zheng, Zining Long, Anding Gao, Jianming Lu, Shuo Wang, Chuanfan Zhong, Houhua Lai, Yufei Guo, Ke Wang, Chen Fang, Xiangming Mao

**Affiliations:** ^1^ Department of Urology, Zhujiang Hospital, Southern Medical University, Guangzhou, China; ^2^ Department of Urology, Fuqing City Hospital Affiliated to Fujian Medical University, Fuzhou, Fujian, China; ^3^ Department of Laboratory Medicine, Fuqing City Hospital Affiliated to Fujian Medical University, Fuzhou, Fujian, China; ^4^ Department of Urology, The Hospital of Trade-Business in Hunan Province, Changsha, China

**Keywords:** pseudouridylation (ψ), lncRNA, biochemical recurrence, prognostic model, prostate cancer

## Abstract

**Background:** Prostate cancer (PCa) is one of the most common cancers in males around the globe, and about one-third of patients with localized PCa will experience biochemical recurrence (BCR) after radical prostatectomy or radiation therapy. Reportedly, a proportion of patients with BCR had a poor prognosis. Cumulative studies have shown that RNA modifications participate in the cancer-related transcriptome, but the role of pseudouridylation occurring in lncRNAs in PCa remains opaque.

**Methods:** Spearman correlation analysis and univariate Cox regression were utilized to determine pseudouridylation-related lncRNAs with prognostic value in PCa. Prognostic pseudouridylation-related lncRNAs were included in the LASSO (least absolute shrinkage and selection operator) regression algorithm to develop a predictive model. KM (Kaplan-Meier) survival analysis and ROC (receiver operating characteristic) curves were applied to validate the constructed model. A battery of biological cell assays was conducted to confirm the cancer-promoting effects of RP11-468E2.5 in the model.

**Results:** A classifier containing five pseudouridine-related lncRNAs was developed to stratify PCa patients on BCR and named the “ψ-lnc score.” KM survival analysis showed patients in the high ψ-lnc score group experienced BCR more than those in the low ψ-lnc score group. ROC curves demonstrated that ψ-lnc score outperformed other clinical indicators in BCR prediction. An external dataset, GSE54460, was utilized to validate the predictive model’s efficacy and authenticity. A ceRNA (competitive endogenous RNA) network was constructed to explore the model’s potential molecular functions and was annotated through GO (Gene Ontology) and KEGG (Kyoto Encyclopedia of Genes and Genomes) pathway analyses. RP11-468E2.5 was picked for further investigation, including pan-cancer analysis and experimental validation. Preliminarily, RP11-468E2.5 was confirmed as a tumor promoter.

**Conclusion:** We provide some evidence that pseudouridylation in lncRNA played a role in the development of PCa and propose a novel prognostic classifier for clinical practice.

## 1 Introduction

According to the cancer statistics in the United States in 2022, prostate cancer alone will account for 27% (268,490/983,160) of cancer diagnoses in men, exceeding lung cancer to be the top one (Siegel et al., 2022). On the other hand, PCa was the second most diagnosed worldwide, only behind lung cancer (Siegel et al., 2021; Sung et al., 2021). Generally, localized PCa patients can yield a favorable prognosis after radical prostatectomy (RP) or radiation therapy (RT). However, biochemical recurrence (BCR), recognized as a detectable serum prostate-specific antigen (PSA) elevation within 10-year follow-ups, occurs in one-third of patients with RP or RT (Pound et al., 1999; Freedland et al., 2007; Boorjian et al., 2011; Van den Broeck et al., 2019). Furthermore, a long-term follow-up BCR study reported that about 24% of patients with BCR developed clinical progression, and the cancer-related mortality hit approximately 16% (Boorjian et al., 2011; Van den Broeck et al., 2019). Therefore, predicting the probability of developing BCR appears pivotal to the prognosis of PCa patients with the increasing incidence rate of PCa.

Thanks to the general application of next-generation sequencing to whole genomes and transcriptomes, numerous pieces of evidence show that less than 2% of the human genome encodes proteins while the rest is transcribed into non-coding RNAs (ncRNAs) (Djebali et al., 2012). Genetic mutations are primarily responsible for cancer, and most of the mutations reside inside the regions that transcribe ncRNAs (Huarte, 2015). In particular, more-than-200-nucleotide long non-coding RNAs (lncRNAs) take up a giant population of ncRNAs, and remarkably, they are gaining more and more attention in the cancer paradigm for exerting dual functions as both oncogenic and tumor-suppressive factors (Sánchez and Huarte, 2013). Given that lncRNAs are reportedly tissue-specific, it is likely that they may share some specific connections with certain cancer subtypes, shedding light on the development of novel biomarkers for the diagnosis, prognosis, or therapeutic targets of cancers (Ling et al., 2015). For instance, prostate cancer antigen 3 (PCA3), the first FDA-approved lncRNA, appears as a promising and pragmatic biomarker for supporting PCa diagnosis (Sartori and Chan, 2014; Sánchez-Salcedo et al., 2021).

RNA modifications are gradually coming into focus due to the development of novel modification detection methods and the realization that ncRNAs are no longer “junks” in the genome and their expression links to complex physiological and pathological processes (Ling et al., 2015; Barbieri and Kouzarides, 2020). Like DNA and proteins, RNAs can be subject to over 170 post-transcriptional modifications, catalyzed by highly conserved enzymes whose dysregulation leads to a broad spectrum of illnesses, including cancer (Jonkhout et al., 2017; Dinescu et al., 2019; Wiener and Schwartz, 2021). Among all these RNA modifications, seven kinds connect to cancer pathogenesis the strongest, such as 7-methylguanosine modification (m7G), N6-methyladenosine modification (m6A), N1-methyladenosine modification (m1A), 5-methylcytosine modification (m5C), pseudouridylation (ψ) and so forth but the underlying machinery of these modifications except m6A in the cancer field, has remained opaque (Barbieri and Kouzarides, 2020). Of the seven ones, pseudouridylation was the first discovered in the 1950s, once called the “fifth RNA nucleotide” (Davis and Allen, 1957) and the most abundant modification in total RNA of human cells (Penzo et al., 2017; Barbieri and Kouzarides, 2020). Pseudouridine used to be frequently detected and studied in tRNA, rRNA, and snRNA (small nuclear RNA); until recently, it was also discovered in mRNA and lncRNA, especially cancer-related lncRNA (Song and Yi, 2017; Dinescu et al., 2019). For example, ψ sites appeared in lncRNAs such as MALAT1 (metastasis-associated lung adenocarcinoma transcript one), XIST (X-inactive specific transcript), TERC (telomerase RNA component), SNHG1 (Small nucleolar RNA host gene one), ZFAS1 (Zinc finger antisense one), etc. Each of them is related to different malignant processes. Unfortunately, there is no established relationship between ψ modification and cancer events, and further studies are required to confirm this correlation. No previous study has revealed the value of ψ-related lncRNAs in PCa. As a result, in this study, we attempted to develop a ψ-related lncRNA predictive model to serve BCR-risk stratification in PCa patients, validate it internally and externally, and investigate its effects on cancer progression using preliminary experiments.

## 2 Materials and methods

### 2.1 Data processing

For bioinformatics analysis, TCGA (The Cancer Genome Atlas) dataset for prostate adenocarcinoma (PRAD) with patients’ clinical data (*n* = 547; tumor tissue = 495, normal tissues = 52) was obtained from the TCGA website (https://portal.gdc.cancer.gov/), and only patients with biochemical recurrent time >1 month (*n* = 460) were included in the survival analyses. Additionally, the GSE54460 dataset (*n* = 100) was acquired from the Gene Expression Omnibus (GEO) database (http://www.ncbi.nlm.nih.gov/geo/). The baseline information for both datasets is deposited in Supplementary Table S1. And we processed the data following the instructions in one article (Li et al., 2021). FPKM (Fragments Per Kilobase Million) data was first transformed into TPM (Transcript Per Million) form and then normalized through log2 (TPM +1). We included R software (version: 4.1.0) and two website tools, “Sangerbox 3.0” (http://vip.sangerbox.com/) and “GEPIA2” (http://gepia2.cancer-pku.cn/), for analyses in the study.

### 2.2 Identification of pseudouridine-related lncRNAs

From literature mining (Rong et al., 2021), 13 pseudouridine-related genes were collected. Then, the expression data of these 13 genes and all lncRNAs from the TCGA-PRAD dataset was extracted. In addition, Spearman’s correlation analysis (de Winter et al., 2016) was employed to look into the relationship between lncRNAs and the 13 Ψ-related genes (criteria: |Spearman R| > .4 and *p* < .05). Eventually, 265 lncRNAs were qualified (Supplementary Table S2). Next, univariate Cox regression analysis (Cox, 1972) was performed on these 265 lncRNAs to evaluate their prognostic values, and finally, 100 lncRNAs with *p*-value <.05 stood out (Supplementary Table S2).

### 2.3 Construction and validation of the Ψ-related predictive model

The LASSO (Least Absolute Shrinkage and Selection Operator) regression (Tibshirani, 1996) algorithm with ten-fold cross validation and penalty (R package “glmnet”) was applied to narrow down the number of genes for establishment of the predictive model. The algorithm constructed different models by including various numbers of Ψ-related lncRNAs (*n* = 100), and the minimum criteria chose the penalty parameter (λ). Ultimately, a five-gene model with the best performance was selected and named the “Ψ-lnc score”. The Ψ-lnc score comes from the formula:

$$\Psi -lnc\;score=\Sigma_{i=1\;}^{N}\left({Coefficient}_{i}\times Expression\;level\;of\;{lncRNA}_{i}\right)$$

Where “N” (N = 5) represents the total number of the lncRNAs in the predictive model, “Coefficient_i_” denotes a specific lncRNA’s coefficient, and “Expression level of lncRNA_i_” refers to the relative expression level of a certain lncRNA.

The TCGA PCa patients were separated into two balanced subsets (the training subset and the testing subset, each number = 230) using the createDataPartition function in R, and the specific Ψ-lnc score for every patient was calculated using the formula above. Given the median scores in the subsets (.296 in the training subset and .288 in the testing subset), the low- and high- Ψ-lnc score subgroups were defined. The Kaplan–Meier (KM) survival analysis (Kaplan and Meier, 1958; Kim et al., 2018; Bichindaritz, 2021; Bichindaritz et al., 2021) in the “survminer” package depicted the BCR-free survival probability curves between the subgroups. The “survivalROC” package drew the 12-, 36-, and 60-month ROC (Receiver Operating Characteristic) curves (Mandrekar, 2010) to evaluate the predictive power of Ψ-lnc score, and the AUCs (Area Under the Curve) of Ψ-lnc score and typical clinicopathological traits were calculated to compare their clinical value. The GSE54460 dataset (N = 100) validated the predictive model externally.

### 2.4 Construction of ceRNA network and functional enrichment analysis

The “GDCRNAtools” package was introduced to help construct the potential competitive endogenous RNA (ceRNA) network (Salmena et al., 2011; Li et al., 2018), and the website tool, “Sangerbox 3.0” (http://vip.sangerbox.com/), conducted the functional enrichments of the mRNAs included in the ceRNA network mentioned above.

### 2.5 Cell culture, RNA extraction, and RT-qPCR assays

Two PCa cell lines, LNCaP and C4-2B, were acquired from the BeNa Culture Collection. Subsequently, both cell lines were cultured in RPMI-1640 media. In addition, 10% fetal bovine serum and 1% Penicillin-Streptomycin solution are combined to make the culture media. The cultivation temperature was 37°C, and the concentration of CO_2_ was 5%. Total RNAs from LNCaP and C4-2B cells were extracted using Trizol reagent (15596018, Takara), and they were then reverse-transcribed into cDNA with the help of TransScript All-in-one First-Strand cDNA Synthesis SuperMix for qPCR (AT341-01, TransGen). RT-qPCR (Real-time quantitative PCR) assays were carried out using the PerfectStart Green (AQ601-02, TransGen) on an Applied Biosystems 7,500 Real-Time PCR System. Eventually, the relative expression of RP11-468E2.5 and other four lncRNAs (GAS1RR, RP11-400K9.4, RP11-400K9.3, and LINC02688) were calculated using glyceraldehyde 3-phosphate dehydrogenase (GAPDH) as the reference. All the experiments were equipped with three replicates. Supplementary Table S7 shows the primers for RP11-468E2.5, GAS1RR, RP11-400K9.4, RP11-400K9.3, and LINC02688.

### 2.6 Patient samples

Prostate cancer tissues (*n* = 10) and benign prostatic hyperplasia tissues (*n* = 10) were collected, respectively, from patients of Zhujiang Hospital, Southern Medical University. Fresh tissues were viewed and approved by two pathologists, frozen immediately in liquid nitrogen, and stored at −80°C.

### 2.7 RNA interference and loss of function assays

GenePharm Company synthesized siRNAs targeting RP11-468E2.5. RT-qPCR confirmed the transfection efficiency after the transfection of siRNAs along with siRNA-Mate (GenePharm) for 72 h. The CCK-8 (Cell Counting Kit-8, MA0218-5, Meilunbio) cell viability assay and colony formation assay inspected the proliferative ability of PCa cell lines after knocking down RP11-468E2.5. The transwell assay examined the change in the invasiveness of PCa cells with downregulation of RP11-468E2.5. Detailed procedures for the above assays are accessible in our previous study (Zhong et al., 2021). All experiments were performed in triplicates. siRNAs targeting sites in RP11-468E2.5 are in Supplementary Table S7.

### 2.8 Statistical analyses

All bioinformatics analyses were performed by R software version 4.1.0 (The R Project for Statistical Computing, Vienna, Austria). The Spearman’s correlation analysis analyzed the correlation between the Ψ-related regulators and lncRNAs. The “survival” package carried out KM survival analysis, and the “survminer” package performed Cox regression analysis. GraphPad Prism 7.0 (GraphPad, La Jolla, CA, United States) analyzed the results of RT-qPCR and CCK-8 cell viability assays. We displayed all statistical results in mean ± SD (standard deviation) with a two-sided test and regarded the results with a *p*-value of less than .05 as statistically significant.

## 3 Results

### 3.1 The landscape of pseudouridylation-related modulators in PCa

**FIGURE 1:**
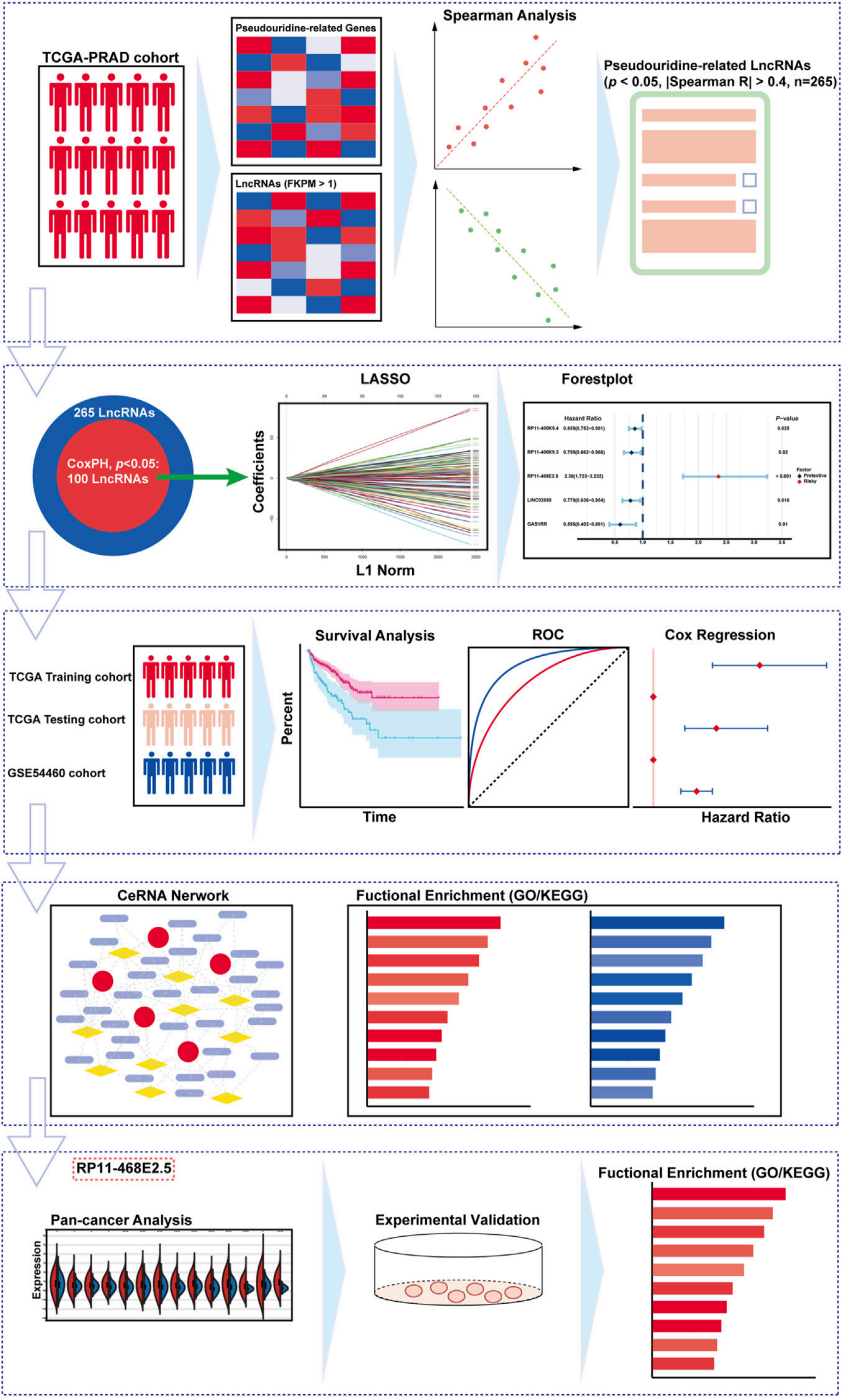
The overall design and the flowchart of the study.

**FIGURE 2:**
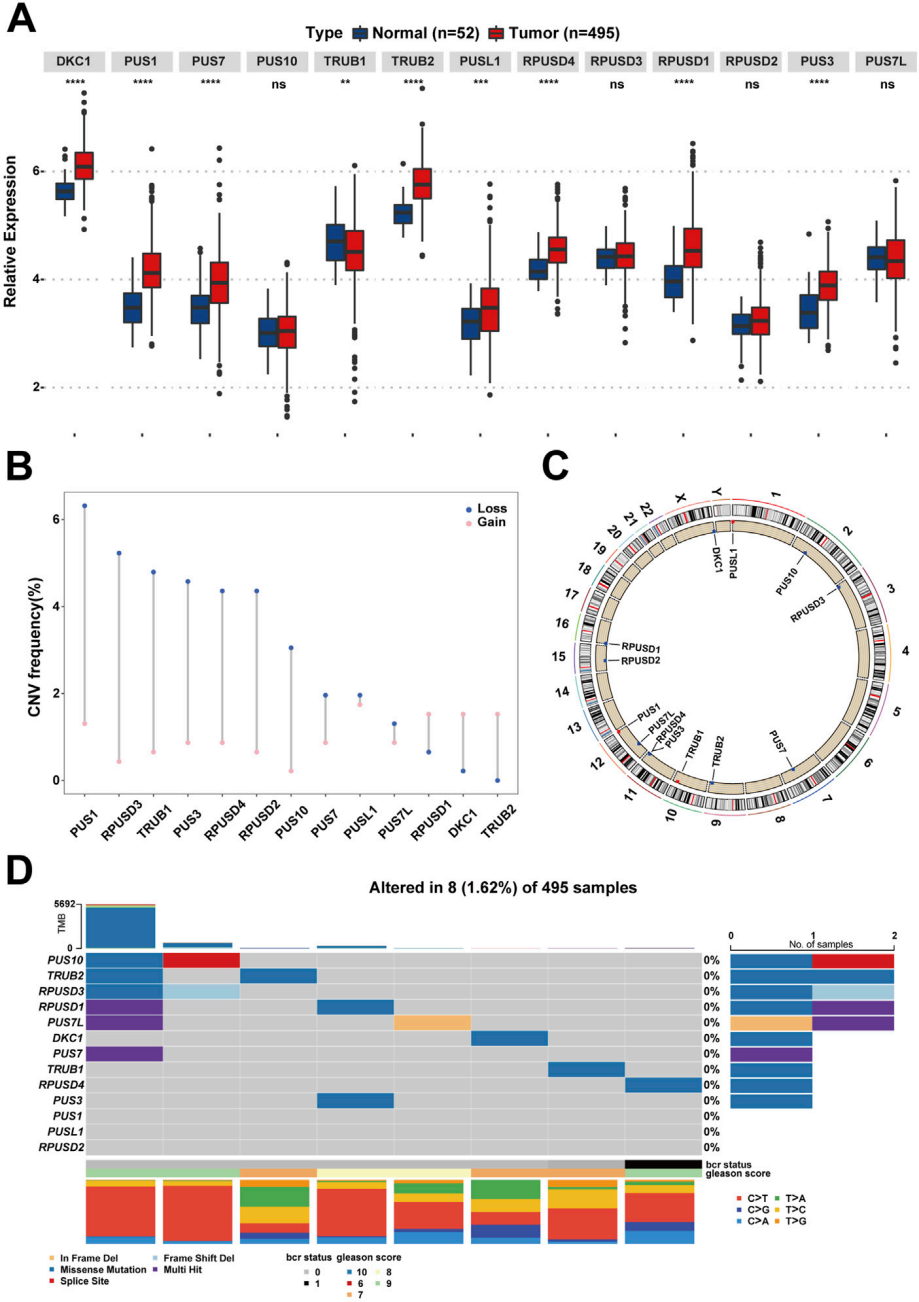
The landscape of Ψ-related regulators on expression, mutation, and chromosome location in PCa. **(A)** The differences of gene expression of the 13 Ψ-related regulators between tumor tissues and adjacent normal tissues in the TCGA-PRAD cohort. Ns, no significance; **p < .01; ***p < .001; ****p < .0001. **(B)** The CNV frequency diagram of the 13 Ψ-related regulators. The two endpoints of each column correspond to two CNV values of a specific Ψ-related gene, with a blue point representing the depletion (Loss) frequency and a pink point denoting the amplification (Gain) frequency, respectively. **(C)** The exact mutation locations of the 13 Ψ-related regulators on chromosomes. **(D)** Eight of 495 (1.62%) PCa patients appeared genetic alterations in the 13 Ψ-related regulators, most of which were missense mutations. The percentages (0%) on the right indicate the mutation frequencies of each Ψ-related regulator, respectively. Each column represents an Ψ gene-mutated individual.

The workflow diagram is displayed in Figure 1. Initially, a pseudouridylation-related gene list (PUS1, RPUSD3, TRUB1, PUS3, RPUSD4, RPUSD2, PUS10, PUS7, PUSL1, PUS7L, RPUSD1, DKC1, and TRUB2) was generated *via* literature mining, and then their expression profiling in the TCGA dataset for prostate adenocarcinoma (TCGA-PRAD) was investigated. As shown in Figure 2A, most of the pseudouridylation-related molecules (8 out of 13) were significantly upregulated in tumor samples (*n* = 492) compared to normal ones (*n* = 52). Then the CNV (copy number variation) mutation data in these genes was examined (Figure 2B). Notably, CNV depletion exists in the majority of them (PUS1, RPUSD3, TRUB1, PUS3, RPUSD4, RPUSD2, PUS10, PUS7, PUSL1, and PUS7L), whereas CNV amplification is prevalent in three of them (RPUSD1, DKC1, and TRUB2). Moreover, Figure 2C depicted the locations of these genes with CNV mutations on chromosomes. In line with this, the somatic mutations of these molecules in PCa were determined using an R package called “maftools.” As a result, only 8 (1.62%) of 495 samples experienced genetic mutations of these genes (Figure 2D). The missense mutation accounts for a giant proportion, followed by multi-hit mutation, in-frame deletion, frame-shift deletion, and splice-site mutation.

### 3.2 Establishment of the prognostic model with pseudouridylation-related LncRNAs and its association with clinical characteristics in PCa

The expression profile of all lncRNAs in TCGA-PRAD was extracted to ascertain the lncRNAs associated with pseudouridylation in PCa. Spearman’s correlation analysis then defined the pseudouridylation-related lncRNAs as ones whose correlation coefficients exceed |.4| with a *p*-value less than .05. Consequently, we obtained 265 pseudouridylation-related lncRNAs (Supplementary Table S1). Next, univariate Cox proportional hazards regression was applied to figure out which lncRNAs presented prognostic value in PCa among these 266 lncRNAs. Consequently, 100 out of 265 lncRNAs appeared to be the prognostic ones (Supplementary Table S2). Subsequently, using the *createDataPartition* function in R, the TCGA-PRAD dataset with 460 samples were divided into two balanced subsets: one training subset and one testing subset, both of which contained 230 patients, respectively. In the training set, the LASSO regression with ten-fold cross validation and penalty was applied to determine the most appropriate prognostic model, using the 100 pseudouridylation-related lncRNAs above (Supplementary Figure S1A). And finally, a five-gene model was considered the most suitable one based on the LASSO results (Supplementary Figure S1B). Following that, the relationship between clinical characteristics and the expression of the five molecules was revealed in the form of a heatmap. Patients with high expression of RP11-468E2.5 (ENSG00000259321) tended to experience advanced T stage, high Gleason scores (GS), BCR, and lymph node metastasis (Supplementary Figure S1C). To further confirm our preliminary discovery, the samples were separated into several binary subgroups based on the GS (GS <= 7; GS > 7), N stage (N0; N1), T stage (T1/2; T3/4), etc. (Supplementary Figure S2). To begin with, patients with GS > 7 expressed more RP11-468E2.5 than those with GS <= 7 (*p* < .001); in contrast, patients with GS > 7 expressed the other four lncRNAs less (Supplementary Figure S2B). Aside from GS, patients in the N-stage and T-stage subgroups had the same expression patterns for RP11-468E2.5 (*p* < .05) and the other four lncRNAs (Supplementary Figures S2C,D). Next ten pairs of samples from local patients with PCa or benign prostatic hyperplasia (BPH) corroborated the difference in expression of these five lncRNAs between tumor (*n* = 10) and benign tissues (*n* = 10) (Supplementary Figure S2E). The expression disparity of four lncRNAs except for LINC02688 between tumor and benign prostate tissues was consistent with the findings above.

### 3.3 Performance and validation of the predictive model with the pseudouridylation-related LncRNAs

**FIGURE 3:**
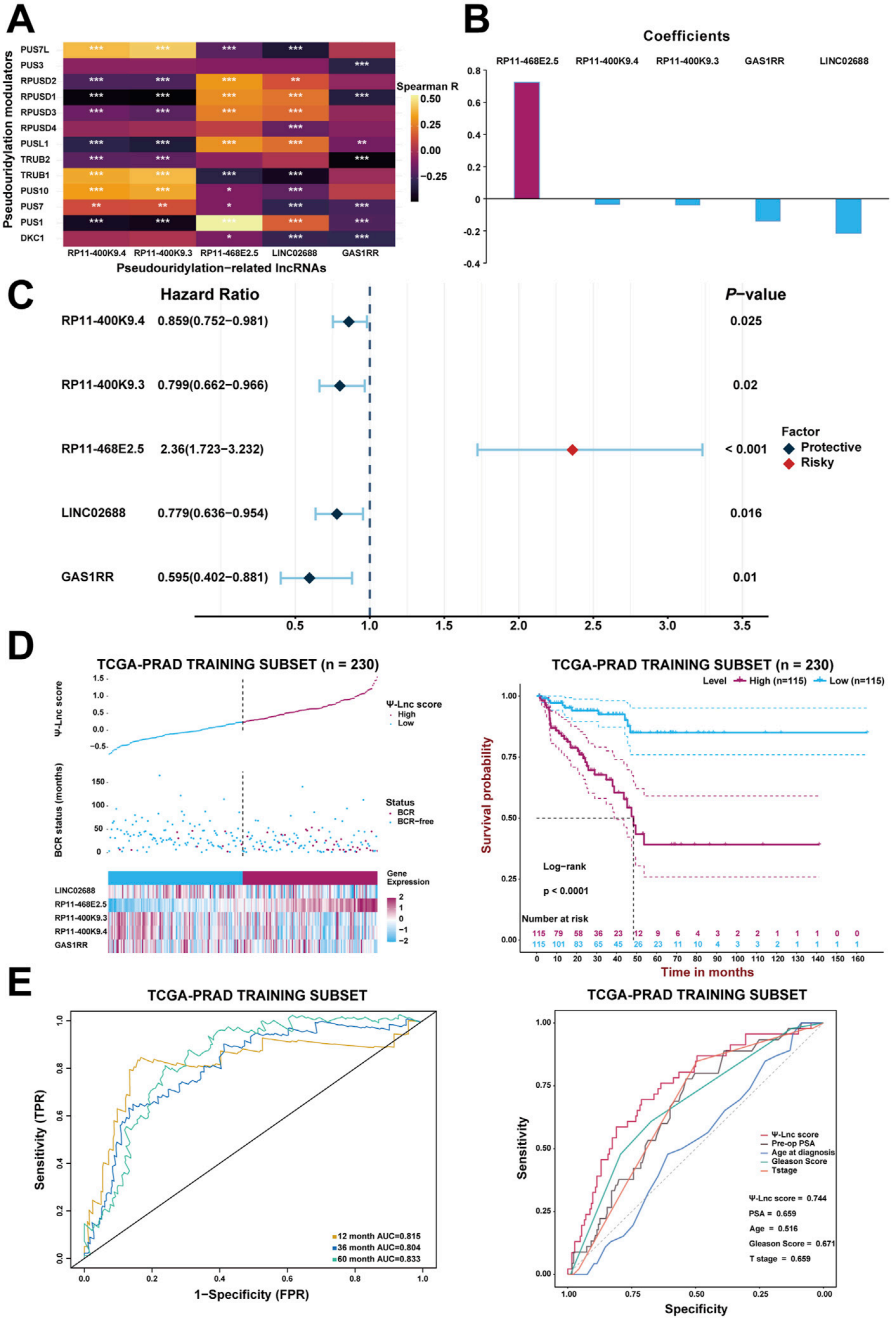
Construction of the prognostic model with Ψ-related lncRNAs. **(A)** The heatmap demonstrates the correlation between the 13 Ψ genes and the five lncRNAs included in the model. *p < .05; **p < .01; ***p < .001. **(B)** The coefficients of each selected lncRNA in the Ψ-lnc scoring formula. **(C)** The forest plot shows the univariate Cox regression results of the five lncRNAs. **(D)** The first diagram depicts PCa patients’ profiles on three aspects, Ψ-lnc score, BCR status, and the five genes’ expression levels, in the TCGA training subset. The second one shows the Kaplan-Meier BCR survival analysis between two Ψ-lnc score subgroups in the training subset. **(E)** The ROC curves show the accuracy of the Ψ-lnc score in predicting BCR-free survival, and the Ψ-lnc score outperforms other clinical indicators.

After generating the predictive model, Spearman’s correlation analysis confirmed the association between the 13 pseudouridylation-related genes and the five pseudouridylation-related lncRNAs and it was presented in the form of a correlation heatmap; generally, a strong correlation showed up between these two subgroups of genes (Figure 3A). Given the LASSO results, a scoring formula based on the weighted expressions of the five chosen genes for scoring every PCa patient’s prognosis was determined and named the “Ψ-lnc score.” The weighted coefficients for each lncRNA were also displayed in a histogram (Figure 3B). In addition, univariate Cox regression analysis confirmed the prognostic value of these lncRNAs, and then the results were exhibited in a forest plot (Figure 3C). Notably, RP11-468E2.5 appeared to be the only risk factor with a hazard ratio (HR) of 2.36 (CI: 1.723–3.232), whereas the others were all protective variables. Subsequently, KM survival curve analysis were introduced to confirm the effects of their expression on PCa prognosis (Supplementary Figures S3A–E). Consistent with the results above, patients with high expression of RP11-468E2.5 had unfavorable BCR-free survival (*p* < .001); in contrast, patients with high expression of each of the other four lncRNAs experienced better BCR-free survival (*p* < .05).

Initially, every patient in the training subset was scored using the formula mentioned above; then, the median score served as the cutoff point to define the high-score and low-score groups inside the training subset. Consequently, Figure 3D depicts the distribution of the Ψ-lnc score, BCR status, expression of the five genes for two subgroups, and the survival analysis. Graphically, more patients who experienced BCR and showed highly-expressed RP11-468E2.5 were in the high-score group than those in the low-score group. In terms of survival, patients in the high-score group had a lower rate of BCR-free survival than those in the low-score group (*p* < .0001). Following that, ROC analysis was employed to draw the 1-year, 3-year, and 5-year ROC curves, calculating the corresponding AUCs to scrutinize the model’s clinically predictive capability (Figure 3E). Remarkably, the five-gene predictive approach showed promise in predicting BCR prognosis in PCa patients (1-year AUC = .815; 3-year AUC = .804; 5-year AUC = .833). In parallel, a multivariate ROC analysis confirmed the feasibility of the model in clinical practice. Compared with some clinical traits like preoperative PSA, age at diagnosis, GS and T stage in BCR prognosis, the Ψ-lnc score outperformed them with its AUC ranking first (.744; AUC_GS_ = .671, AUC_PSA_ = .659, AUC_T stage_ = .659, AUC_Age_ = .516). Additionally, two Cox regression models (the univariate and multivariate ones) were employed to investigate the clinical value of Ψ-lnc score and the aforementioned clinicopathological features (Supplementary Figure S4). Consequently, Ψ-lnc score surpassed all other features with the highest HR both in univariate and multivariate Cox regression analysis.

**FIGURE 4:**
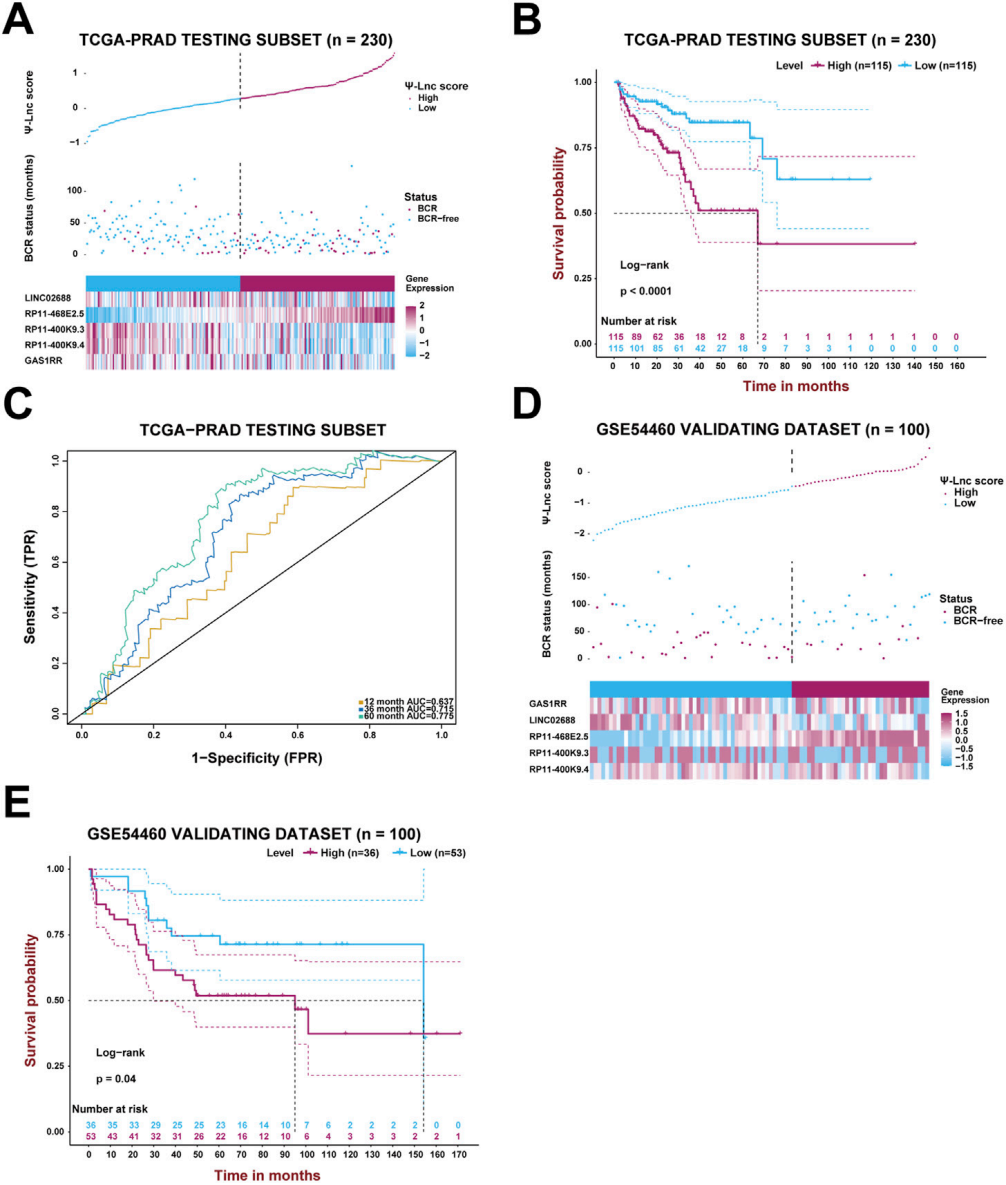
Validation of the prognostic model with Ψ-related lncRNAs. **(A)** The PCa patients’ profiles on three aspects, Ψ-lnc score, BCR status, and the five genes’ expression levels, in the TCGA testing subset. **(B)** The Kaplan-Meier BCR survival analysis between two Ψ-lnc score subgroups in the testing subset. **(C)** The ROC curves show the accuracy of the Ψ-lnc score in predicting BCR-free survival. **(D)** The PCa patients’ profiles on three aspects, Ψ-lnc score, BCR status, and the five genes’ expression levels, in the GSE54460 validating dataset. **(E)** The Kaplan-Meier BCR survival analysis between two Ψ-lnc score subgroups in the GSE54460 validating dataset.

Likewise, the established model was then internally validated with the TCGA-PRAD testing subset. After separating the testing subset into the high-score and low-score groups based on the median Ψ-lnc score, the analyses above were repeated to verify the model’s authenticity. Figure 4A displays the Ψ-lnc score distribution, BCR status, and gene expression profiles in the two groups. Figure 4B shows that patients in the low-score group yielded more favorable BCR-free survival outcomes than those in the high-score group (*p* < .0001), consistent with the previous results. In terms of predictive power, the model’s 12-month, 36-month, and 60-month AUCs in the testing subset are .637, .715, and .775, respectively, harboring considerable outcomes (Figure 4C). Finally, the GSE54460 dataset was introduced to inspect the model’s external validity (Figures 4D,E). Patients in the high-score and low-score groups showed a significant difference in BCR-free survival; high-score patients yielded worse outcomes than low-score ones.

### 3.4 Construction of the potential competing endogenous RNA network and functional enrichment analysis

**FIGURE 5:**
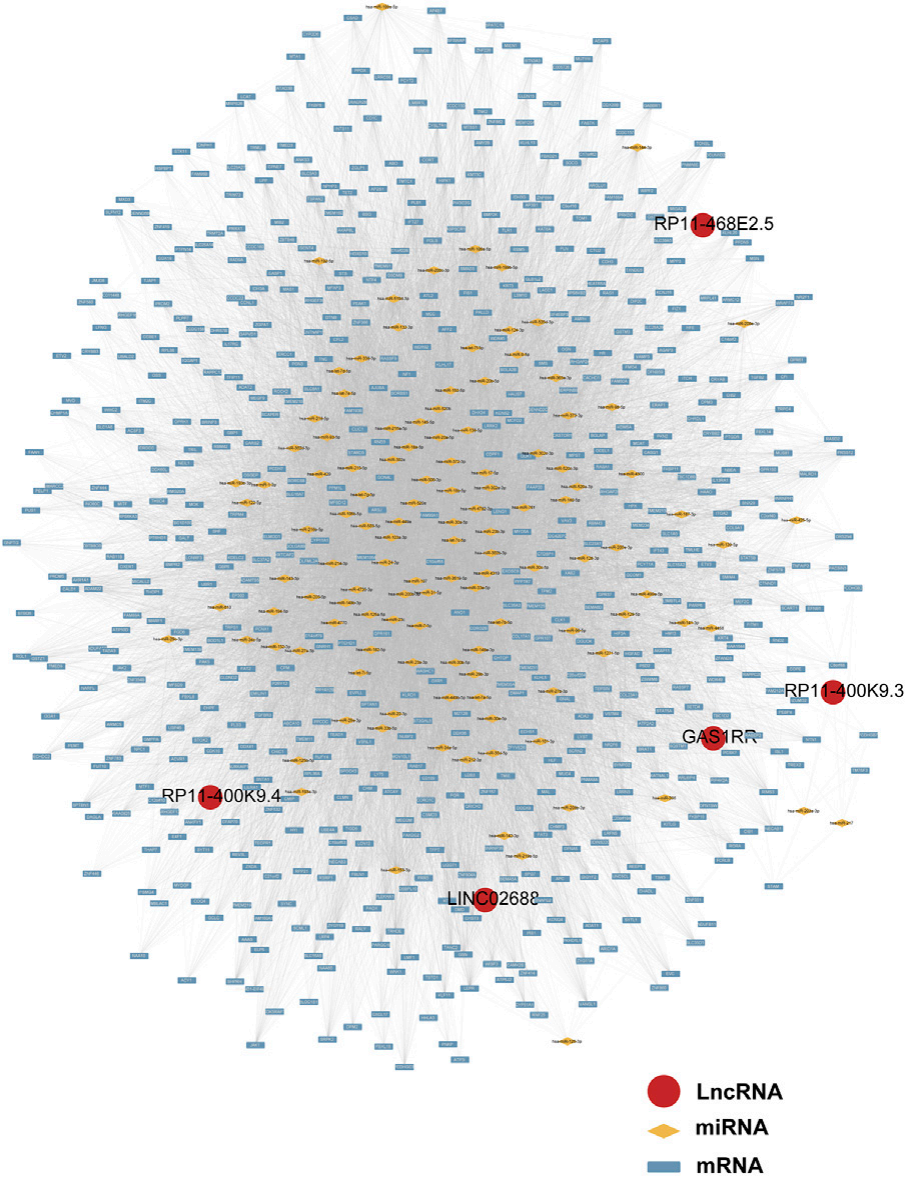
The ceRNA network of five lncRNAs with potential miRNAs and mRNAs.

**FIGURE 6:**
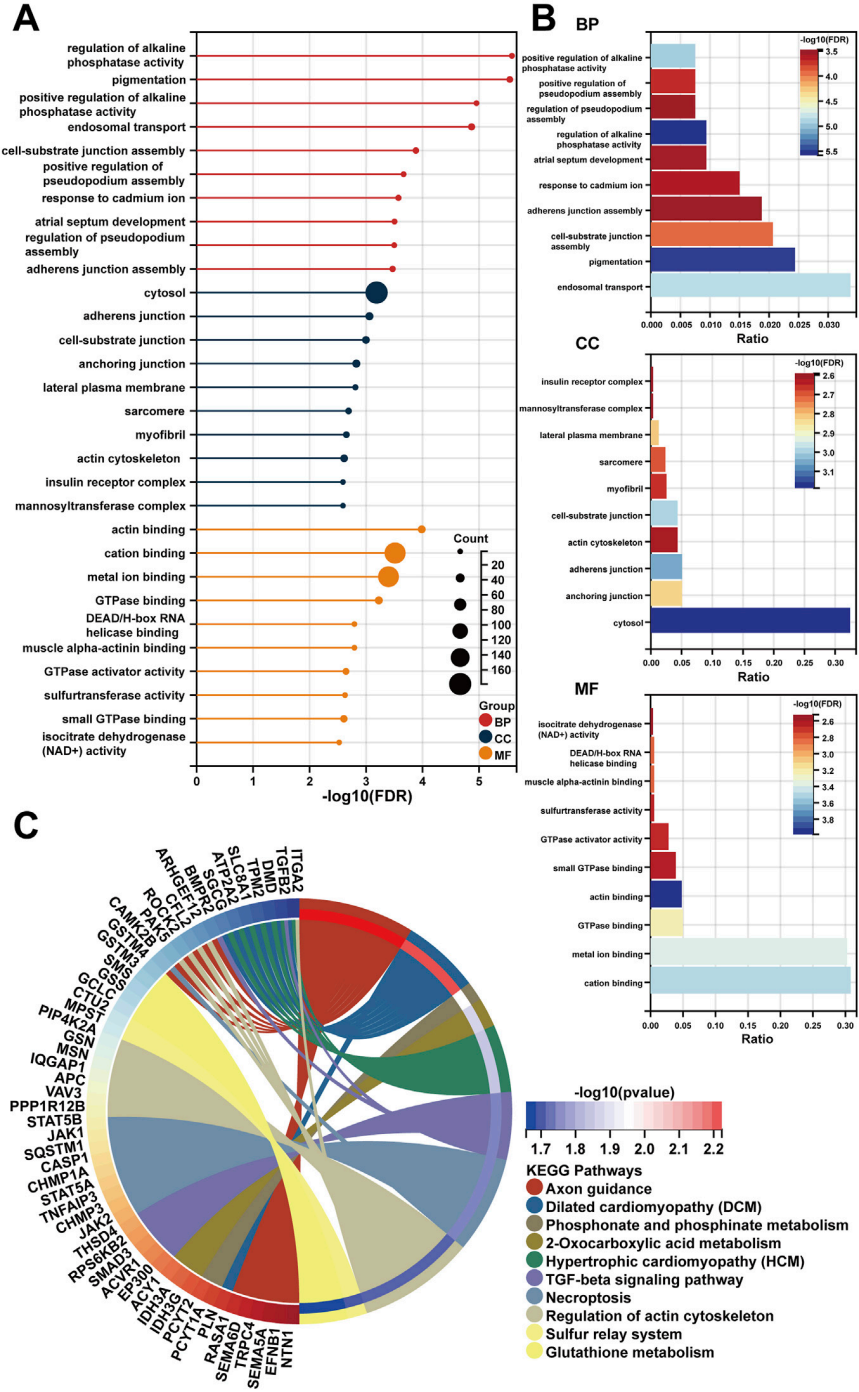
The functional enrichment analysis of 628 mRNAs in the ceRNA network. **(A,B)** The diagrams show the top 10 terms in three parts (BP, CC, and MF) of the GO analysis. **(C)** The top 10 pathways in the KEGG analysis.

Following a preliminary examination of the predictive model’s performance, attention was drawn to the molecular functions that these genes may possess. It is well known that lncRNAs are likely involved in the ceRNA network to exert their effects. Thus, the processed expression data from the TCGA-PRAD dataset was utilized to explore the potential ceRNA network with the help of an R package called “GDCRNAtools.” Given the results, all the lncRNA-miRNA-mRNA pairs with their *p*-values and correlation coefficients were obtained. The pairs above were then filtered under the inclusive conditions (*p* < .05 and |correlation coefficients| > .4) to form the ceRNA network. As a result, a ceRNA network of 754 molecules (5 lncRNAs, 121 microRNAs, and 628 mRNAs) was identified and then visualized using the software “Cytoscape” (Figure 5). Red circles indicate the five lncRNAs, yellow lozenges represent the 121 microRNAs, and blue rectangles represent the 628 mRNAs in the diagram. Detailed links among these three elements are available in Supplementary Table S3. Later, the 628 mRNAs were put into functional enrichment analysis to further investigate their potential roles in biological processes. And the website tool called “Sangerbox 3.0” was applied to carry out the enrichment analyses, revealing the gene ontology (GO) terms and KEGG (Kyoto Encyclopedia of Genes and Genomes) pathways highly related to these genes. The GO terms with *p* < .05 and FDR (false discovery rate) < .25 were considered significant; the KEGG pathways with *p* < .05 were also considered meaningful. On the one hand, the top 10 GO terms from each of the three categories (BP, Biological Process; CC, Cellular Component; MF, Molecular Function) were chosen to exhibit in Figures 6A,B. In particular, attention was paid to the underlying biological processes. The top 10 GO terms in BP are regulation of alkaline phosphatase activity (GO:0010692), pigmentation (GO:0043473), positive regulation of alkaline phosphatase activity (GO:0010694), endosomal transport (GO:0016197), cell-substrate junction assembly (GO:0007044), positive regulation of pseudopodium assembly (GO:0031274), response to cadmium ion (GO:0046686), atrial septum development (GO:0003283), regulation of pseudopodium assembly (GO:0031272), and adherens junction assembly (GO:0034333). On the other hand, the top 10 KEGG pathways were also displayed in the form of a ring plot as shown in Figure 6C, including axon guidance (hsa04360), dilated cardiomyopathy (DCM) (hsa05414), phosphonate and phosphinate metabolism (hsa00440), 2-oxocarboxylic acid metabolism (hsa01210), hypertrophic cardiomyopathy (HCM) (hsa05410), TGF-beta signaling pathway (hsa04350), necroptosis (hsa04217), regulation of actin cytoskeleton (hsa04810), sulfur relay system (hsa04122), and glutathione metabolism (hsa00480). The complete information about the GO and KEGG analyses is in Supplementary Table S4.

### 3.5 Pan-cancer analysis and experimental validation of RP11-468E2.5’s effects on PCa

RP11-468E2.5 was the only risk factor with an HR of 1.86 in the established model (Figure 3C), indicating its cancer-promoting effects, so we decided to study its role in cancer, especially PCa, further. At the beginning, a pan-cancer analysis was performed to explore the relationship between its expression and tissue type (normal, tumor) and between its expression and cancer prognosis. Sangerbox 3.0 was used with TCGA data to determine the expression difference of RP11-468E2.5 between tumor-adjacent and tumor samples in each type of tumor, and unpaired Wilcoxon Rank Sum and Signed Rank Tests was implemented to analyze the significance of the difference. Consequently, RP11-468E2.5 is up-regulated significantly (*p* < .05) in fourteen types of tumors such as PRAD, LUAD (Lung adenocarcinoma), COAD (Colon adenocarcinoma), COADREAD (Colon adenocarcinoma/Rectum adenocarcinoma), ESCA (Esophageal carcinoma), STES (Stomach and Esophageal carcinoma), KIRP (Kidney renal papillary cell carcinoma), KIRC (Kidney renal clear cell carcinoma), KIPAN (Pan-kidney cohort; KICH, Kidney Chromophobe; KIRC; KIRP), STAD (Stomach adenocarcinoma), HNSC(Head and Neck squamous cell carcinoma), LIHC (Liver hepatocellular carcinoma), BLCA (Bladder urothelial carcinoma), and CHOL (Cholangiocarcinoma), as shown in Supplementary Figure S5A. Next, the Cox proportional hazards regression model analyzed the relationship between RP11-468E2.5’s expression and the prognosis of each tumor, one by one. Then the Log-rank test was run to obtain prognostic significance. Finally, the high expression of RP11-468E2.5 in the three types of tumors (PRAD; LUSC, Lung squamous cell carcinoma; ACC, Adrenocortical carcinoma) shows a poor prognosis while the low expression level of RP11-468E2.5 in another four types of tumors (PAAD, Pancreatic adenocarcinoma; SKCM, Skin Cutaneous Melanoma; BLCA; READ) indicates a poor prognosis (Supplementary Figure S5B). Therefore, RP11-468E2.5 is upregulated generally in tumors and its expression demonstrates dual effects on cancer patients’ prognosis.

**FIGURE 7:**
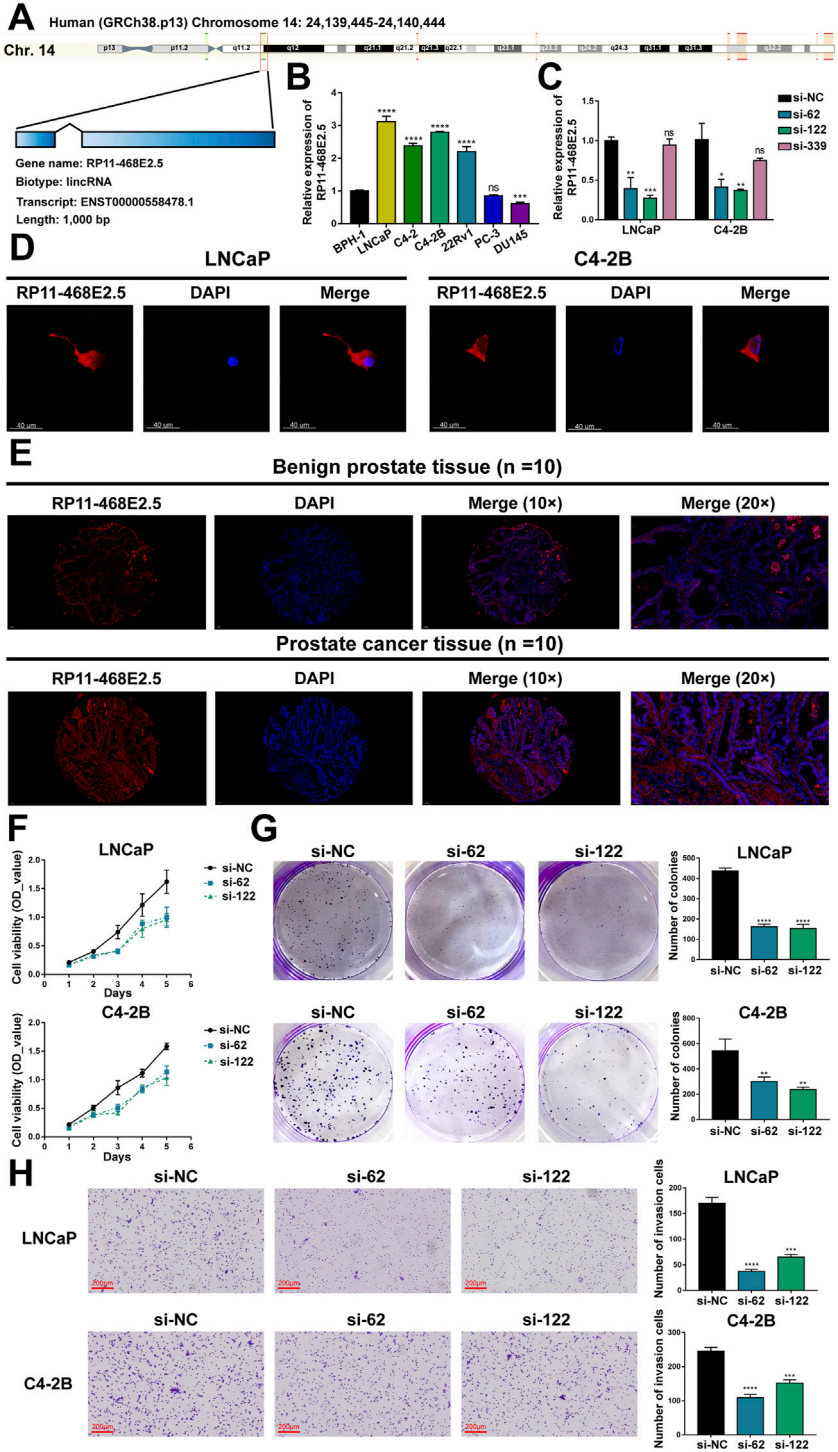
Experimental validation of RP11-468E2.5’s cancer-promoting effects on PCa. **(A)** The gene information of RP11-468E2.5. **(B)** The basal expressions of RP11-468E2.5 in six PCa cell lines and one normal prostate cell line (BPH-1). **(C)** The gene-silencing efficiencies of three siRNAs in LNCaP and C4-2B PCa cell lines. **(D)** The fluorescence *in situ* hybridization (FISH) assays illustrated that RP11-468E2.5 mainly exists in the cytoplasmic part of LNCaP and C4-2B cell lines. **(E)** FISH assays confirmed that RP11-468E2.5 is highly expressed in tumor tissue compared to benign prostate tissue. **(F)** The proliferation (CCK-8) assays showed silencing RP11-468E2.5 compromised cell viability in LNCaP and C4-2B cell lines. **(G)** The plate colony formation assays demonstrated downregulating RP11-468E2.5 attenuated cell viability in LNCaP and C4-2B cell lines. **(H)** The transwell assay showed silencing RP11-468E2.5 hampered PCa cells’ invasiveness.

Then experiments were performed to confirm RP11-468E2.5’s role in PCa. To begin with, detailed information on RP11-468E2.5 was scrutinized (Figure 7A). RP11-468E2.5 is a lncRNA of 1,000 bp, located on Chromosome 14: 24,139,445–24,140,444. The basal expression of RP11-468E2.5 was checked in six PCa cell lines and one normal prostate cell line (Figure 7B). As a result, RP11-468E2.5 is highly-expressed in four out of six PCa cell lines (LNCaP, C4-2, C4-2B, and 22Rv1) compared to the normal prostate cell line, BPH-1. Thus, two cell lines with the highest expression levels of RP11-468E2.5, LNCaP and C4-2B, were selected for further research. As shown in Figure 7C, three si-RNAs (si-62, si-122, and si-339) were designed to interrupt the expression of RP11-468E2.5 in LNCaP and C4-2B; however, only si-62 and si-122 silenced RP11-468E2.5 significantly, compared to the control group, si-NC. Fluorescence *in situ* hybridization (FISH) assays showed that RP11-468E2.5 mainly exists in the cytoplasmic part of LNCaP and C4-2B cell lines (Figure 7D). Furthermore, its subcellular localization was confirmed in tissues collected from patients with PCa or BPH (Figure 7E). Consistent with our previous findings, RP11-468E2.5 appears highly expressed in the tumor tissue compared to benign prostate tissue. Then the CCK-8 assay examined whether the two RP11-468E2.5-silenced cell lines’ proliferative ability was attenuated. After the 5-day observation, silencing RP11-468E2.5 slowed PCa cells’ proliferation significantly (Figure 7F). In another aspect, plate colony formation assay was performed to investigate the influence of knocking down RP11-468E2.5 on PCa cells’ proliferation ability. Consequently, knock-down of RP11-468E2.5 imposed an attenuative effect on PCa cell viability, too (Figure 7G). The transwell assay demonstrated the decreased invasiveness of PCa cells after downregulating RP11-468E2.5 (Figure 7H). Silencing RP11-468E2.5 hindered PCa cells’ invasive ability. Taken together, RP11-468E2.5 was preliminarily confirmed to act as a promoting factor in the development of PCa.

### 3.6 Functional enrichment analysis for RP11-468E2.5

**FIGURE 8:**
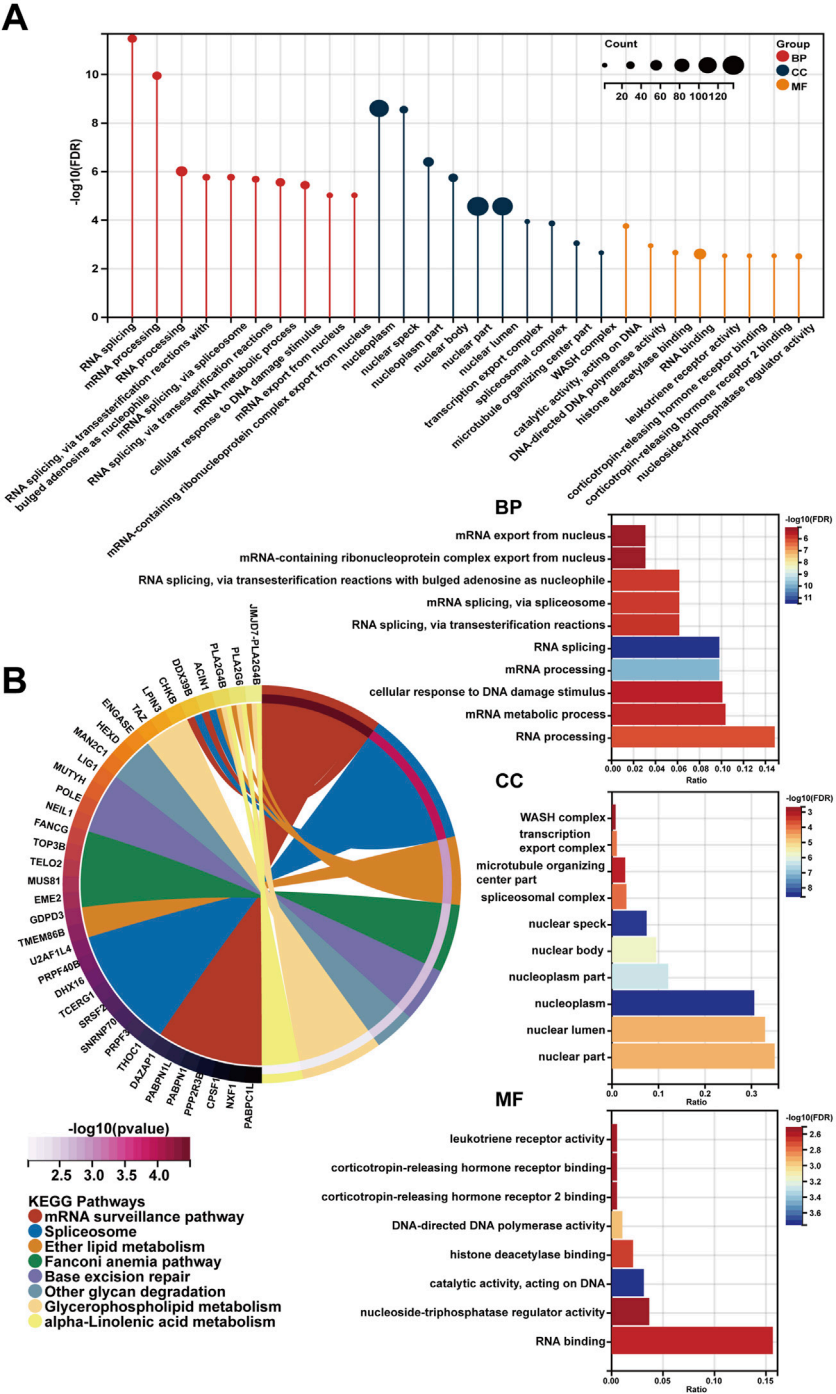
The functional enrichment analysis of a gene set including RP11-468E2.5 and its similar genes. **(A)** The diagrams show the top 10 terms in three parts (BP, CC, and MF) of the GO analysis. **(B)** The top eight pathways in the KEGG analysis.

In spite of RP11-468E2.5’s cancer-promoting effects on PCa, the underlying mechanism remains opaque. Thus, RP11-468E2.5 and its top 1,000 similar genes (Supplementary Table S5) obtained from the website GEPIA2 were used to perform functional enrichment analysis. Likewise, the GO terms with *p* < .05 (FDR <.25) and the KEGG pathways with *p* < .05 were considered significant. Figure 8 exhibited the 10 GO terms (except for the MF category) and the top eight KEGG pathways. Specifically, the GO terms in the biological process category are as follow: RNA splicing (GO:0008380), mRNA processing (GO:0006397), RNA processing (GO:0006396), RNA splicing, *via* transesterification reactions with bulged adenosine as nucleophile (GO:0000377), mRNA splicing, *via* spliceosome (GO:0000398), RNA splicing, *via* transesterification reactions (GO:0000375), mRNA metabolic process (GO:0016071), cellular response to DNA damage stimulus (GO:0006974), mRNA export from nucleus (GO:0006406), and mRNA-containing ribonucleoprotein complex export from nucleus (GO:0071427). And the top eight KEGG pathways are as foloow: mRNA surveillance pathway (hsa03015), Spliceosome (hsa03040), Ether lipid metabolism (hsa00565), Fanconi anemia pathway (hsa03460), Base excision repair (hsa03410), Other glycan degradation (hsa00511), Glycerophospholipid metabolism (hsa00564), and alpha-Linolenic acid metabolism (hsa00592). These results may shed some light on the RP11-468E2.5’s molecular functions. Detailed information about the functional enrichment results is in Supplementary Table S6.

## 4 Discussion

The “central dogma” has become the consensus in molecular biology for a drastically long period; The biological diversity all comes from the changes in the nucleotide sequences in DNA/RNA and the 64 codons together to determine the amino acid sequences (Boriack-Sjodin et al., 2018). With techniques for sequencing RNA and DNA pioneered by Fred Sanger in the 1960s and 1970s (Brownlee et al., 1967; Sanger et al., 1977), scientists have been gradually gaining access to the biological details inside these macromolecules. Simultaneously, the effects of chemical modifications to DNA and post-translational modifications to proteins on gene regulation and cancer biology have gained incredible attention in the research community (Esteller, 2007; Chen et al., 2017). Despite this, our understanding of an intermediate layer of regulation between DNA and proteins is still relatively limited. As numerous RNA modifications have come to light, they collectively constitute the concept of “epitranscriptome” (Saletore et al., 2012). These modifications regulate almost every aspect of RNA, such as splicing, nuclear export, translation, degradation, and so on (Gilbert et al., 2016; Peer et al., 2017). It is becoming clear that RNA functioning depends on RNA modifications greatly. And with the dysregulation of RNA epigenetic processes come common human diseases, including cancer (Esteller and Pandolfi, 2017; Barbieri and Kouzarides, 2020). Pseudouridylation is one kind of cancer-associated internal RNA modification but is still rarely investigated in the cancer field compared to two notable ones, m6A, and m5C chemical modifications (Esteller and Pandolfi, 2017; Barbieri and Kouzarides, 2020; Nombela et al., 2021). Pseudouridylation is reportedly the most abundant modification in ncRNAs, and previous studies confirmed its existence in tRNA, rRNA, and snoRNAs. But with the birth of various Ψ-Seq techniques, pseudouridine was also observed in lncRNAs such as XIST and MALAT1, and among ncRNAs, lncRNAs possess the highest abundance of pseudouridine (Li et al., 2015; Esteller and Pandolfi, 2017). How pseudouridylation impacts cancer through modulating lncRNA remains to be elucidated.

PCa is responsible for 7% of newly diagnosed malignancies in males worldwide (2021). According to the GLOBOCAN 2020 estimates, Asia accounted for 26.2% of the global PCa incidence rate and 32.1% of its mortality rate in 2020 (Sung et al., 2021). PCa is becoming an unaffordable health issue and an economic burden for the public, even in low-incidence-rate areas like Asia. And indeed, a deeper understanding of PCa is urgent for improving prognosis prediction and offering therapeutic vision. From the academic standpoint, no research on pseudouridine-modified lncRNAs affecting PCa’s carcinogenesis or progression has existed. Therefore, we aim to reveal some details about this novel topic with bioinformatics and preliminary experiments.

Initially, 13 pseudouridine-related modulators (DKC1, PUS1, PUS7, PUS10, TRUB1, TRUB2, PUSL1, RPUSD4, RPUSD3, RPUSD1, RPUSD2, PUS3, and PUS7L) were confirmed for further analysis (Penzo et al., 2017). Next, pseudouridine-related lncRNAs in PCa were identified by performing Spearman’s correlation analysis between the Ψ-related genes and all lncRNAs in the TCGA-PRAD dataset. And a five-pseudouridine-related lncRNA scoring signature for predicting BCR survival in PCa, named “Ψ-lnc score”, was developed by the LASSO approach (Tibshirani, 1996), given that LASSO is broadly introduced to the Cox proportional hazard regression model for survival analysis in the bioscience arena (Tibshirani, 1997; Zhang and Lu, 2007). The LASSO method generated a scoring formula based on the expression levels of the five selected genes, of which RP11-468E2.5 tends to be a risk factor, and the other four (GAS1RR, RP11-400K9.4, RP11-400K9.3, and LINC02688) serve as favorable ones.

RP11-468E2.5 is a lncRNA with a length of 1,000 nucleotides, and its influences on cancer are poorly understood. To date, only one study showed that RP11-468E2.5 could negatively target STAT5 and STAT6 to affect the JAK/STAT signaling pathway indirectly (Darnell et al., 1994; Leonard and O’Shea, 1998). Upregulating RP11-468E2.5 curtails the JAK/STAT signaling pathway by targeting two molecules, STAT5 and STAT6, and finally attenuates cell proliferation but boosts cell apoptosis in colorectal cancer (Jiang et al., 2019). However, how RP11-468E2.5 regulates STAT5 and STAT6 negatively remains to be elucidated. In contrast, LINC02688, one of the protective indicators in the constructed model, stays more poorly studied. Only one study unprecedentedly revealed that LINC02688 was expressed less in gastric cancer (GC) tissues compared to paired adjacent normal tissues, and its expression further decreased when GC developed into an advanced one (Fattahi et al., 2021). Additionally, it preliminarily showed considerable prognostic power in GC based on the AUC values of the ROC curve. Nevertheless, more rigorous studies with more clinical samples of different types of cancers and populations from different genetic backgrounds are necessary to explore the exact role of LINC02688 in cancer progression. Lastly, the other three novel lncRNAs haven’t unveiled their roles in cancer yet.

After the model construction, the predictive accuracy of Ψ-lnc score was then inspected using KM survival analysis and uni-/multi-variate time-dependent ROC analysis (Heagerty et al., 2000). As a result, Ψ-lnc score appeared to be a satisfactory indicator with the highest AUC value, outperforming typical clinicopathological parameters such as PSA, GS, pathological T stage, and so forth. Subsequently, a dataset (GSE54460) was introduced for the model’s external validation; the outcomes were consistent with the previous ones.

Increasing studies demonstrate that lncRNAs that harbor MREs (miRNA-response elements) come up as natural miRNA decoys (Karreth and Pandolfi, 2013). And they are bioinformatically presumed to be broad miRNA targets, suggesting their functioning as ceRNAs (competitive endogenous RNAs) (Griffiths-Jones et al., 2008; Paraskevopoulou et al., 2013). With the ceRNA hypothesis, we asked whether these five lncRNAs in the predictive model work as ceRNAs *via* the R package “GDCRNAtools” and consequently obtained an interactive ceRNA network. To better understand the ceRNA network’s functions, functional annotation analysis (GO analysis and KEGG pathway analysis) was performed. As mentioned before, RP11-468E2.5 was the only risk factor with the highest coefficient in the scoring formula, suggesting its dominant role in the model. Given the pan-cancer analysis, RP11-468E2.5 is highly-expressed (*p* < .05) in fourteen types of tumors, including PCa, compared to their correspondent normal tissues. Additionally, its expression exerts tumor-suppressing or cancer-promoting effects on seven kinds of malignancies. Then *in vitro* experiments were implemented to validate its oncogenic role, and consistent results were found in cell proliferation assays in two PCa cell lines (C4-2B and LNCaP). Furthermore, its molecular functions were annotated bioinformatically; annotation analysis using RP11-468E2.5 and its 1,000 similar genes showed it might be involved in the RNA splicing process.

The current study has its limitations, too. Firstly, more public datasets are necessary for better external validation of the established model. Secondly, more advanced experimental validation of RP11-468E2.5 is meaningful for inspecting its molecular functions for the sake of novel pseudouridine-related biomarker development. In aggregate, the constructed model still has a long way to go before it comes into practice.

## 5 Conclusion

A predictive model containing pseudouridine-related lncRNAs was created to forecast BCR survival probabilities for PCa patients and validated internally and externally. Furthermore, preliminary experiments were performed to validate the cancer-promoting effects of the dominant lncRNA, RP11-468E2.5, in the model. This work sheds some insight into the influence of non-coding RNA modifications on PCa. Still, in-depth studies need to explore how the novel modification, pseudouridylation, functions in the cancer arena.

## Data Availability

The datasets presented in this study can be found in online repositories. The names of the repository/repositories and accession number(s) can be found in the article/Supplementary Material.

## Supplementary material

Presentation 1 Contains Supplementary Figures S1-S5

Data Sheet 1 Contains Supplementary Tables S1-S7

## Glossary

PCa: prostate cancer
RP: radical prostatectomy
RT: radiation therapy
BCR: biochemical recurrence
PSA: prostate-specific antigen
ncRNA: non-coding RNA
lncRNA: long non-coding RNA
m7G: 7-methylguanosine
m6A: N6-methyladenosine
m1A: N1-methyladenosine
m5C: 5-methylcytosine
ψ: pseudouridylation/pseudouridine
MALAT1: metastasis-associated lung adenocarcinoma transcript 1
XIST: X-inactive specific transcript
TERC: telomerase RNA component
SNHG1: small nucleolar RNA host gene 1
ZFAS1: zinc finger anti-sense 1
TCGA: the cancer genome atlas project
TCGA-PRAD: TCGA dataset for prostate adenocarcinoma (TCGA-PRAD)
GEO: gene expression omnibus
FPKM: fragments per kilobase million
TPM: transcript per million
LASSO: least absolute shrinkage and selection operator
KM: Kaplan-Meier
ROC: receiver operating characteristic
AUC: area under the curve
ceRNA: competitive endogenous RNA
GAPDH: glyceraldehyde 3-phosphate dehydrogenase
CCK-8: cell counting kit-8
CNV: copy number variation
GS: Gleason score
BPH: benign prostatic hyperplasia
HR: hazard ratio
GO: gene ontology
KEGG: Kyoto encyclopedia of genes and genomes
BP: biological process
CC: cellular component
MF: molecular function
FISH: fluorescence *in situ* hybridization
GC: gastric cancer
MRE: MiRNA-response element
