# Supplementary material for: A five-pseudouridylation-associated-LncRNA classifier for primary prostate cancer prognosis prediction
Source: Front Genet. 2023 Jan 10;13:1110799. doi: 10.3389/fgene.2022.1110799 (PMC9871836; doi:10.3389/fgene.2022.1110799)
Supplement: Supplementary file 1 [file Presentation1.pdf]

Supplementary Material

A Five-Pseudouridylation-Associated-LncRNA Classifier for Primary Prostate Cancer Prognosis Prediction

Pengxiang Zheng[1,2]†, Zining Long[1]†, Anding Gao[3]†, Jianming Lu[1], Shuo Wang[1], Chuanfan Zhong[1], Houhua Lai[1], Yufei Guo[1], Ke Wang[1,4]§, Chen Fang[2]§, and Xiangming Mao[1]*

*** Correspondence:** XiangmingMao, mxm631221@126.com

§ Co-correspondence: Ke Wang, wangke_225@126.com; Chen Fang, cfang365@hotmail.com

1.1 Supplementary Figures

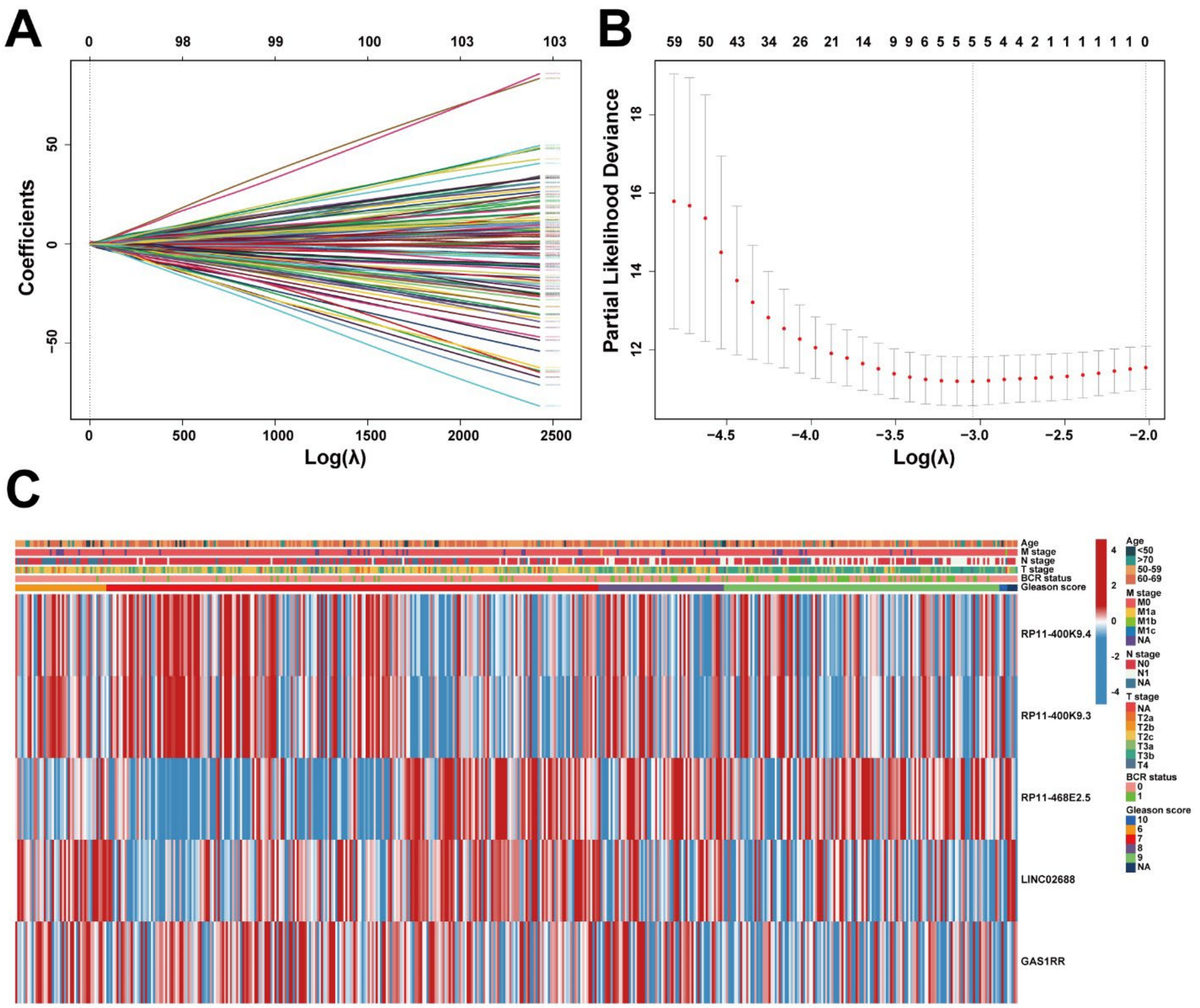

Supplementary Figure 1. (A & B) Construction of the prognostic model with Ψ-related lncRNAs via LASSO algorithm. (C) The heatmap shows the association between the 5 Ψ-related lncRNAs' expression profiles and clinical traits.

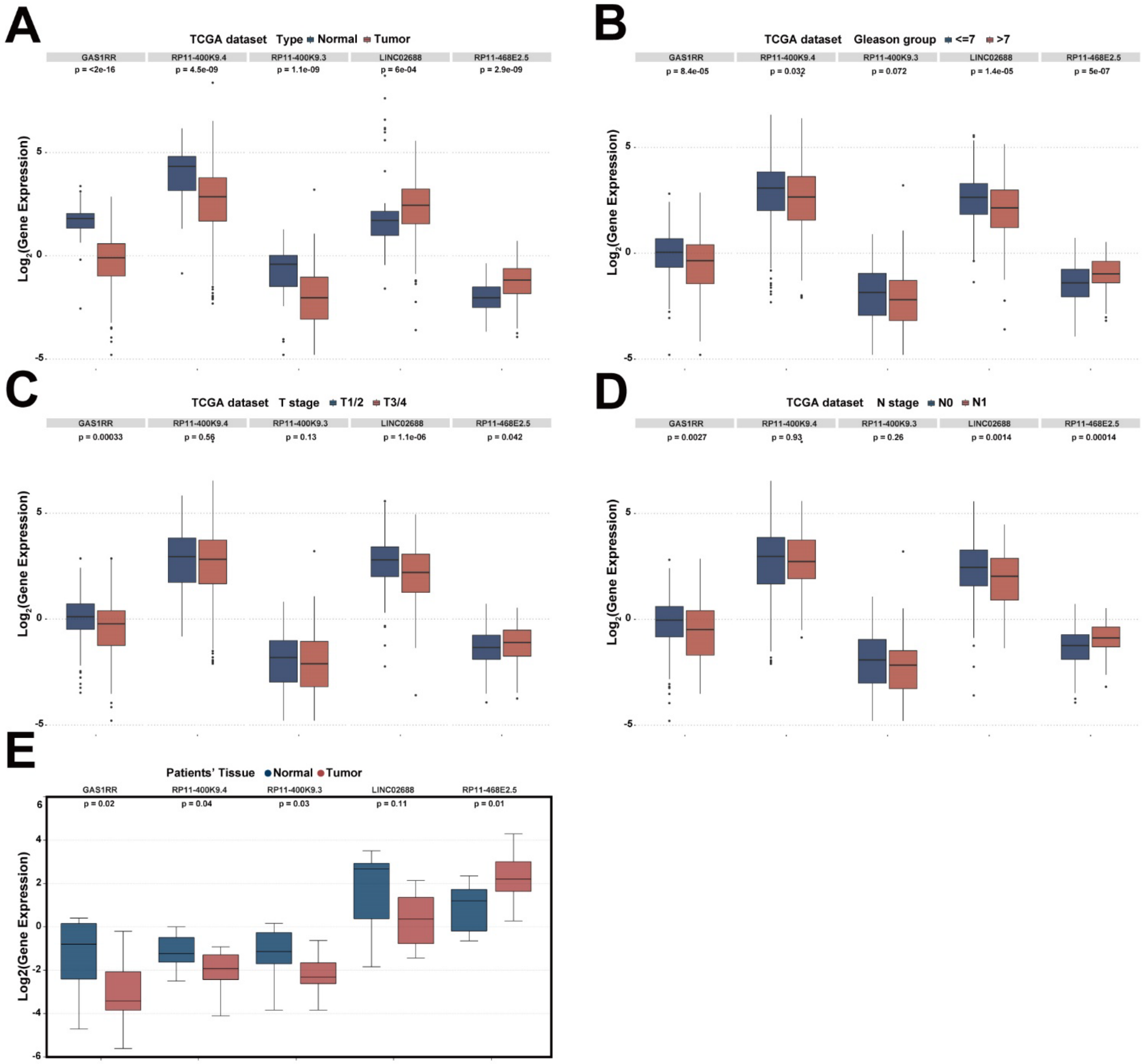

Supplementary Figure 2 The expression landscapes of the 5 Ψ-related lncRNAs between several binary subgroups. (A) The expression landscape of the 5 Ψ-related lncRNAs between tumor tissues and adjacent normal tissues. (B) The expression landscape of the 5 Ψ-related lncRNAs between two GS subgroups (GS <= 7; GS > 7). (C) The expression landscape of the 5 Ψ-related lncRNAs between two T stage subgroups (T1/2; T3/4). (D) The expression landscape of the 5 Ψ-related lncRNAs between two N stage subgroups (N0; N1). (E) The expression landscape of the 5 Ψ-related lncRNAs between tumor and benign prostate tissues collected from local patients with PCa or BPH.

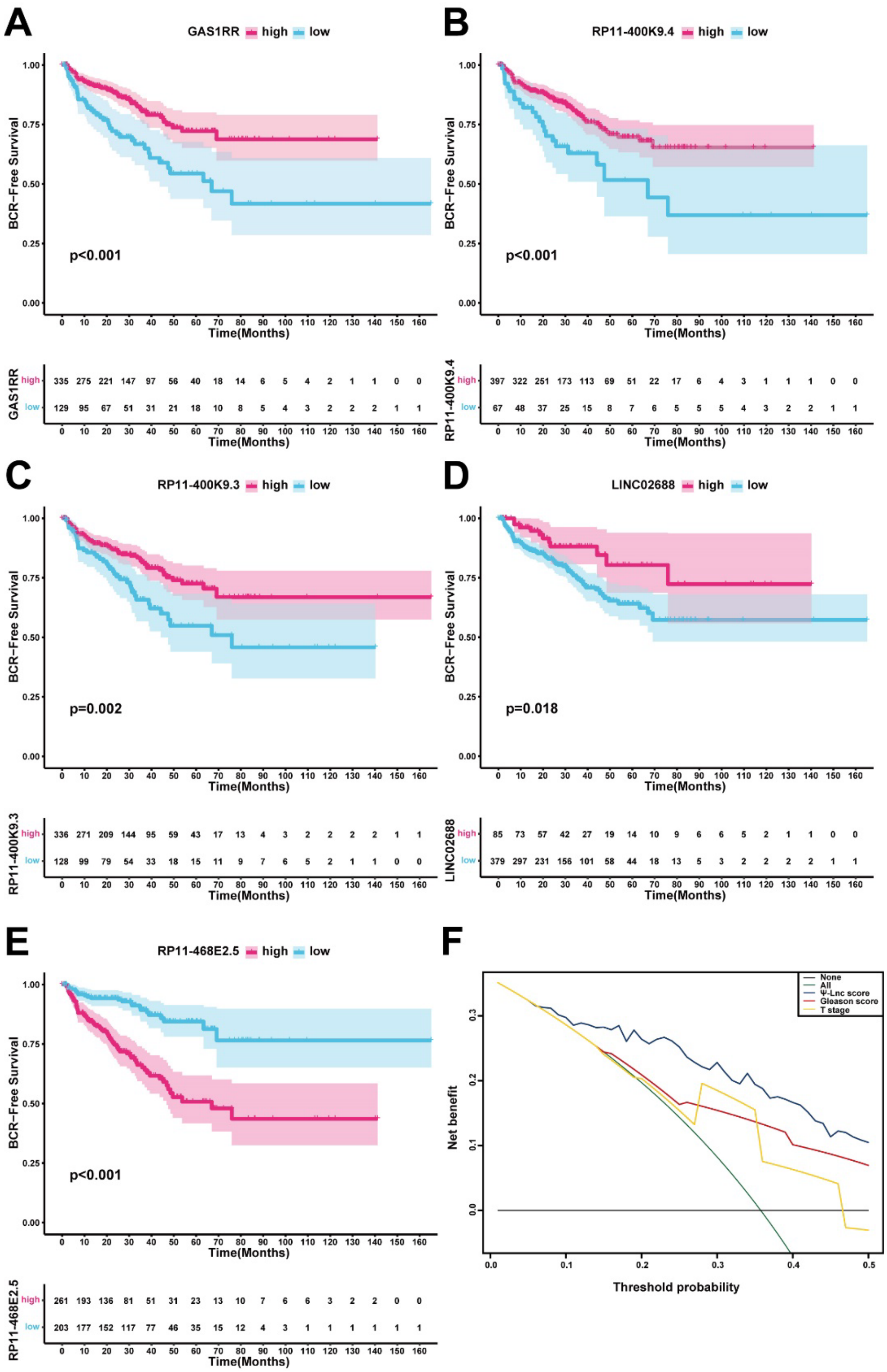

Supplementary Figure 3 The Ψ-related lncRNAs' prognostic value on predicting the BCR-free survival in Ψ-lnc score subgroups in PCa and the benefit analysis on Ψ-lnc score plus other clinical indicators. (A-E) The Kaplan-Meier survival curves of the 5 Ψ-related lncRNAs in Ψ-lnc score subgroups in PCa. (F) The decision curve analysis showed the variation of benefits with different predictive strategies.

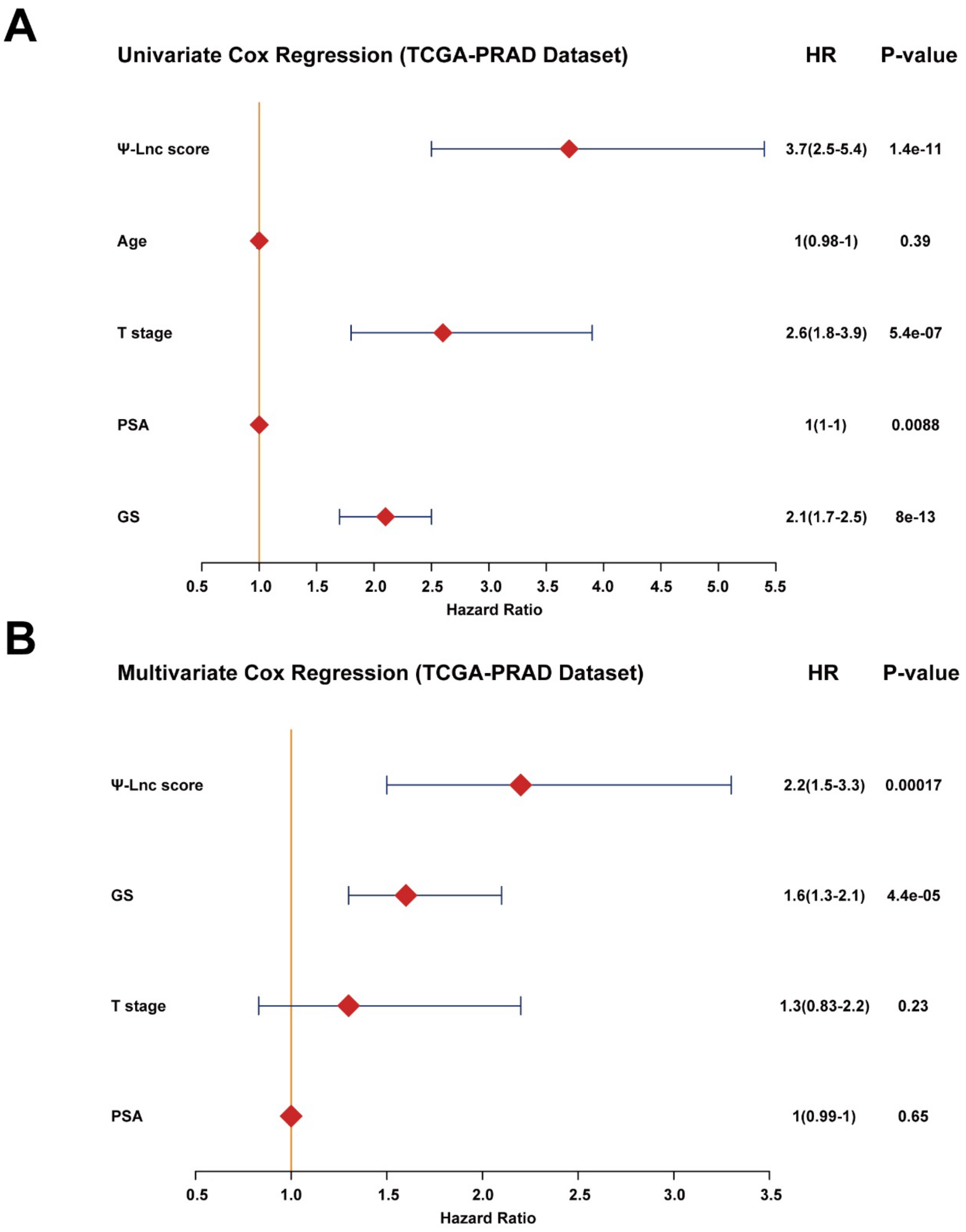

Supplementary Figure 4 Validation of Ψ-lnc score performance using univariable and multivariable Cox analyses in the TCGA-PRAD dataset. (A) The forest plot shows the univariate Cox analysis results for the Ψ-lnc score and other clinical traits. (B) The forest plot shows the multivariate Cox analysis results for the Ψ-lnc score and other clinical traits.

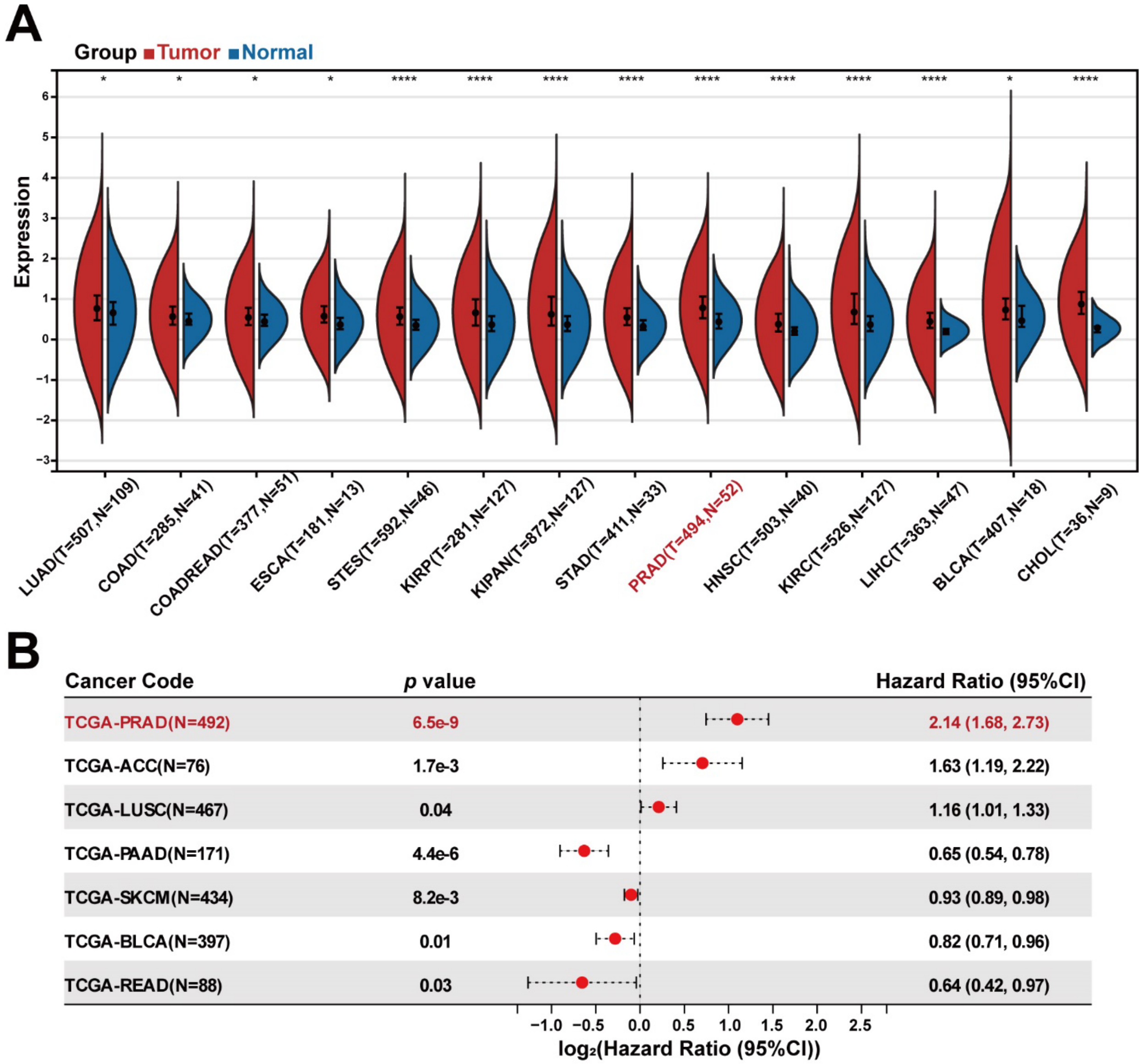

Supplementary Figure 5 Pan-cancer analysis of RP11-468E2.5. (A)The expression landscape of RP11-468E2.5 between tumor and benign tissues in 14 types of cancers (TCGA). (B) The prognostic value of RP11-468E2.5 in 7 kinds of cancers, and RP11-468E2.5 presented as a risk factor in the TCGA-PRAD dataset.
